# Emergence of Invasive Group A Streptococcus Infection in an Infant: A Case Report

**DOI:** 10.5811/cpcem.21172

**Published:** 2024-12-06

**Authors:** Alexis N. Roach, Ryan Kwong, Sarah Sylvester

**Affiliations:** University of Arkansas for Medical Sciences, Arkansas Children’s Hospital, Department of Pediatrics, Section of Emergency Medicine, Little Rock, Arkansas

**Keywords:** Group A streptococcus, Streptococcus pyogenes, osteomyelitis, necrotizing fasciitis

## Abstract

**Introduction:**

Group A streptococcus (GAS) manifests as a spectrum of illnesses, ranging from mild to life-threatening. While relatively rare in infants, GAS infections can present with grave consequences.

**Case Report:**

An eight-month-old infant was found to have GAS bacteremia complicated by sepsis and disseminated intravascular coagulation, resulting in lower extremity myositis and tissue ischemia. Tissue ischemia progressed to dry gangrene requiring below-knee amputation followed by six weeks of antibiotics.

**Conclusion:**

This case serves as a reminder of the critical importance of vigilance, prompt recognition, and aggressive intervention in the management of invasive GAS infections in infants.

## INTRODUCTION

Group A streptococcus (GAS), also known as *Streptococcus pyogenes*, is a widely recognized pathogen responsible for a broad spectrum of illnesses, from pharyngitis and impetigo to life-threatening invasive conditions such as necrotizing fasciitis and streptococcal toxic shock syndrome.^1^–^3^ Prevalence of GAS infections tends to increase with age, although GAS poses a significant public health concern in the pediatric population.^3^,^4^ However, it is uncommon for infants to develop disseminated GAS infection, which can lead to difficulties in diagnosis and treatment.^5^ This case report focuses on the clinical symptoms, treatment, and complications experienced by an eight-month-old male diagnosed with disseminated GAS bacteremia.

## CASE REPORT

An eight-month-old, previously healthy male presented to the emergency department (ED) due to concern for extremity edema. His parents reported initial symptoms of fever, congestion, diarrhea, and increased fussiness that began five days prior to presentation. Two weeks prior to presentation, the infant experienced multiple sores over his nose that bled intermittently with congestion. At that time, his pediatrician prescribed a five-day course of cefdinir, which he completed. The infant’s two older siblings had recent diagnoses of influenza, and his parents had been diagnosed with GAS pharyngitis 2–3 weeks prior to presentation. The infant then began to experience right upper extremity edema one day prior to presentation, which prompted his parents to return to his pediatrician’s office for further evaluation.

In his pediatrician’s office, the infant was diagnosed with GAS pharyngitis, using a rapid GAS test, and prescribed oral amoxicillin-clavulanic acid. At that time, the right upper extremity edema was believed to be secondary to insect bites. That evening, the infant began to experience bilateral lower extremity edema with skin discoloration, decreased oral intake, and increased irritability. Due to continued concern, the infant’s parents brought him to an outside ED where laboratory findings revealed hypoalbuminemia (albumin 2.2 grams per deciliter [g/dL] [2.6–3.6 g/dL]), elevated liver function tests (aspartate aminotransferase (AST) 128 units per liter [U/L] [20–60 U/L]), and leukopenia (3.3 x 10^3^ per μL [6–17.5 x 10^3^ per μL]), therefore, he was transferred to our facility for a higher level of care.

On presentation to our ED, the infant was alert, interactive, non-toxic appearing, tachycardic (heart rate 184 beats per minute), and febrile (38.4 °Celsius). Initial exam revealed edematous right upper arm and forearm, bilateral lower extremity edema, and palpable pulses in all four extremities. He was also noted to have congestion and rhinorrhea. He was hypoglycemic (glucose 50 milligrams [mg]/dL, reference range 70–105 mg/dL) and required a 5 milliliter per kilogram (mL/kg) dextrose 10% bolus. Additional laboratory evaluation revealed metabolic acidosis (pH 7.07, serum bicarbonate 11 millimoles [mmol]/L [18–27 mmol/L]); lactic acid 8.6 mmol/L (0.5–2.2 mmol/L); elevated inflammatory markers (C-reactive protein 245 mg/L [0–9.9 mg/L]); procalcitonin 16.58 nanograms [ng]/mL [0–2 ng/mL])15; elevated AST 128 U/L (20–60 U/L); normal alanine aminotransferase, and mild hypoalbuminemia (albumin 2.5 g/dL [2.6–3.6 g/dL]). Intravenous (IV) vancomycin and ceftriaxone were initiated, and a 20 mL/kg normal saline bolus was administered due to persistent tachycardia, fever, and laboratory findings consistent with sepsis.

Radiographs of the right upper extremity were unremarkable. Surgery was consulted due to concern for limb ischemia. Computed tomography of the right humerus with contrast was concerning for myositis and cellulitis without drainable fluid collection, and an ultrasound of the right upper extremity was without thrombus. However, lower extremity perfusion and edema acutely worsened with absence of Doppler signal in the left lower extremity distal to the popliteal fossa. A second 20 mL/kg normal saline bolus was administered due to overt sepsis. Concern for disseminated intravascular coagulation (DIC) was heightened as additional laboratory findings revealed prolonged prothrombin time (15.7 seconds [9.8–13.3 seconds], leukopenia (4.39 x 10^3^ per μL [6–17.5 x 10^3^ per μL]; bandemia 25% (5–11%), anemia (hemoglobin 8.9 g/dL [10.5–13.5 g/dL], hematocrit 28% [33–39%]); and thrombocytopenia (34 x 10^3^ per μL [150–400 x 10^3^ per μL]).

The infant required admission to the pediatric intensive care unit due the critical nature of his illness. In the setting of DIC with significant thrombocytopenia and anemia, fresh frozen plasma, packed red blood cells, platelets, and cryoprecipitate were given. Antimicrobial coverage was broadened, with continued IV vancomycin and the addition of clindamycin and cefepime. Ultrasound of the left lower extremity revealed venous and arterial thrombi with femoral vein and popliteal artery thrombus. Vascular surgery was consulted, but no revascularization surgical options were identified; therefore, unfractionated heparin was initiated for anticoagulation. Echocardiogram revealed a structurally normal heart with no evidence of intracardiac vegetation. Blood culture obtained in the ED grew Gram-positive cocci in chains at approximately 12 hours, which was noted to be *S pyogenes* on polymerase chain reaction, confirmed on culture speciation. Upon availability of bacterial susceptibilities, antimicrobial coverage was narrowed to ampicillin.

**CPC-EM Capsule**

What do we already know about this clinical entity?

*Group A streptococcus (GAS) infection results in a spectrum of illnesses, ranging from pharyngitis and impetigo to necrotizing fasciitis and toxic shock syndrome*.

What makes this presentation of disease reportable?

*A rare example of invasive GAS in an infant, this case highlights the formidable and rapidly progressive nature of systemic disease*.

What is the major learning point?

*Group A streptococcus infections pose diagnostic challenges in infants, and prompt intervention can attenuate the morbidity associated with systemic complications*.

How might this improve emergency medicine practice?

*Early recognition and aggressive intervention in the management of GAS infections in infants can mitigate progression to invasive infection*.

**Image 1:**
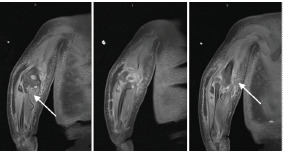
Magnetic resonance imaging of the right upper extremity demonstrating extensive cellulitis, myositis, and fasciitis with no definite or discrete focus of osteomyelitis (arrows).

**Image 2:**
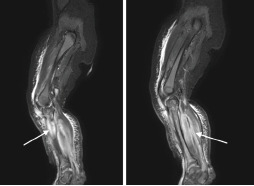
Magnetic resonance imaging of the left lower extremity demonstrating osteomyelitis involving the entire left tibia, the mid/distal left fibula, and the left ilium adjacent to the sacroiliac joint (arrow in left photo). Diffuse muscular edema is visualized in the left gluteal musculature, quadriceps musculature, and extensively involving both the anterior and posterior compartments of the lower leg (arrow in right photo).

The infant required intubation and mechanical ventilation on day 2 of hospitalization due to concern for airway edema in the setting of worsening facial and neck edema with desaturations. He became hypotensive following intubation requiring pressor support and stress-dose hydrocortisone. Due to worsening clinical status, a continuous penicillin infusion was initiated. The infant remained intubated for seven days, and he was quickly weaned to room air following extubation. Magnetic resonance imaging of the right upper extremity revealed three rim-enhancing, soft-tissue collections concerning for abscesses with extensive cellulitis, myositis, and fasciitis surrounding the entire right upper extremity (Image 1). A small elbow joint effusion with synovitis was also noted. The infant underwent irrigation and debridement of the right upper extremity with elbow arthrotomy on day 6 of hospitalization. Orthopedic surgery noted purulence and necrotic tissue during this procedure and collected cultures. On day 9 of hospitalization, he underwent a second irrigation and debridement of the right upper extremity. His left lower extremity ischemia progressed to dry gangrene requiring left below-knee amputation on day 20 of hospitalization. Magnetic resonance imaging of the left lower extremity directly prior to scheduled, below-knee amputation revealed osteomyelitis involving the entire left tibia, mid/distal left fibula, and left ilium adjacent to the sacroiliac joint (Image 2). Below-knee amputation cultures grew *Candida parapsilosis*, *Enterococcus faecalis*, and possible anaerobes. Oral fluconazole was initiated for candida coverage, IV penicillin G provided adequate coverage for *E faecalis*, and oral metronidazole was initiated for anaerobic coverage. The infant completed six weeks of IV penicillin G, oral fluconazole, and oral metronidazole from source control with amputation, and was safely discharged home with continued outpatient therapies.

## DISCUSSION

Group A streptococcus often infiltrates deep tissues through superficial skin lesions, such as an insect bite in the infant described in this case, with inflammation quickly becoming extensive.^3^,^5^,^6^ Bacteremia frequently occurs concurrently leading to rapid decompensation.^1^–^3^,^5^,^6^ Invasive streptococcal pharyngitis is a less common source of infection, but it was also relevant to the infant described in this case.^2^ Appropriate management of GAS complications, including myositis and myonecrosis, osteomyelitis, toxic shock syndrome, and necrotizing fasciitis, requires a multidisciplinary approach to management

Collaboration with medical and surgical subspecialties, including infectious diseases, hematology, vascular surgery, and orthopedic surgery, was necessary to coordinate and enhance inpatient management. Ancillary therapy with physical therapy, occupational therapy, and speech therapy assisted the infant in wound care and continued therapeutic mobility measures. Integrated patient-centered care largely contributed to mitigation of disease progression and the ability to successfully discharge home with continued outpatient management.

## CONCLUSION

This case highlights the formidable nature of invasive group A streptococcus infections in infants, illustrated by the rapid progression to life-threatening sepsis, DIC, and limb ischemia necessitating amputation. Despite advances in medical care, GAS infections continue to pose significant challenges, often presenting with rapid progression and severe systemic manifestations, as demonstrated in this case. Early recognition and initiation of appropriate antimicrobial therapy are paramount to mitigate the progression of disease and improve outcomes. Additionally, vigilance for complications such as DIC and limb ischemia is crucial, as prompt intervention can attenuate morbidity and mortality associated with these sequelae. By sharing our clinical experience and insights, we hope to contribute to the collective knowledge base and ultimately improve clinical outcomes for infants afflicted by this life-threatening condition.
